# Effect of K^+^ Force Fields on Ionic Conductivity and Charge Dynamics of KOH in Ethylene Glycol

**DOI:** 10.1021/acs.jpcb.3c08480

**Published:** 2024-04-04

**Authors:** Amey Thorat, Rohit Chauhan, Rohan Sartape, Meenesh R. Singh, Jindal K. Shah

**Affiliations:** † School of Chemical Engineering, Oklahoma State University, Stillwater, Oklahoma 74078, United States; ‡ Department of Chemical Engineering, University of Illinois at Chicago, Chicago, Illinois 60608, United States

## Abstract

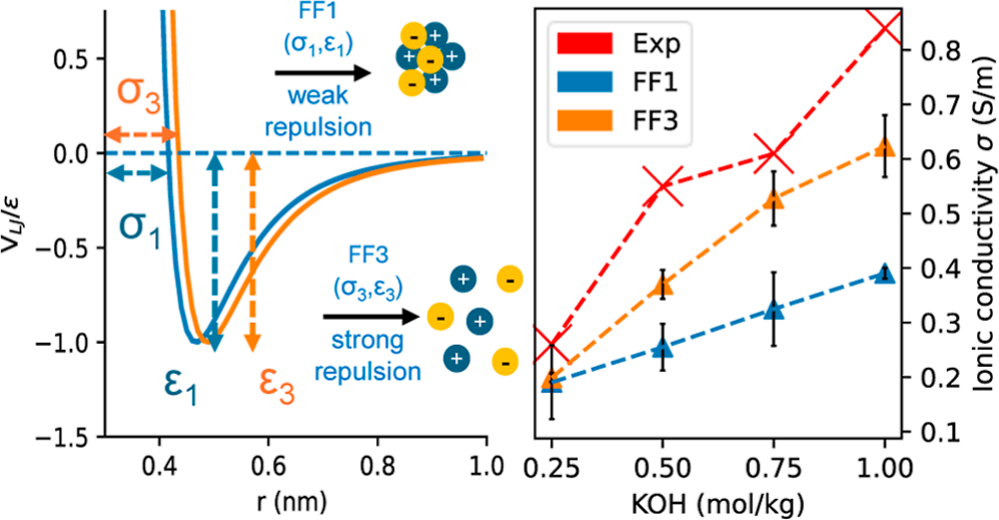

Predicting ionic conductivity is crucial for developing efficient electrolytes for energy storage and conversion and other electrochemical applications. An accurate estimate of ionic conductivity requires understanding complex ion–ion and ion–solvent interactions governing the charge transport at the molecular level. Molecular simulations can provide key insights into the spatial and temporal behavior of electrolyte constituents. However, such insights depend on the ability of force fields to describe the underlying phenomena. In this work, molecular dynamics simulations were leveraged to delineate the impact of force field parameters on ionic conductivity predictions of potassium hydroxide (KOH) in ethylene glycol (EG). Four different force fields were used to represent the K^+^ ion. Diffusion-based Nernst–Einstein and correlation-based Einstein approaches were implemented to estimate the ionic conductivity, and the predicted values were compared with experimental measurements. The physical aspects, including ion-aggregation, charge distribution, cluster correlation, and cluster dynamics, were also examined. A force field was identified that provides reasonably accurate Einstein conductivity values and a physically coherent representation of the electrolyte at the molecular level.

## Introduction

Various physical, chemical, and electrochemical processes have evolved for capturing CO_2_ in an effort toward mitigating climate change.^1,2^ Many of these processes leverage the reactive chemistry between alkali hydroxides and CO_2_ to produce carbonates and bicarbonates.^3^ A recently developed, migration-assisted moisture gradient (MAMG) process^4^ is an electrochemical process that uses a mixture of KOH and ethylene glycol (EG) to capture CO_2_. In this process, the electrolyte serves two purposes: capturing CO_2_ via reaction with hydroxide ions to form bicarbonates and facilitating ion transport within the system under the influence of an electric field. The CO_2_ capture reaction is favored in a medium with hydrogen bonding capability,^5,6^ but the presence of any moisture leads to the formation of carbonate and CO_2_ and hence is detrimental to the overall efficiency of the process.^7^ Therefore, it is necessary to employ a nonaqueous hydrogen bonding solvent such as EG. A similar mixture of EG and KOH-based solvents has been previously explored as low temperature-transition mixtures.^8^ This creates a scope for improving the process efficiency by tailoring the formulation, possibly by incorporating other high CO_2_ solubility solvents such as ionic liquids (ILs) into the electrolyte.^9−11^

Understanding ion aggregation and the degree of dissociation is also important to map ion availability for the CO_2_ capture reaction. These factors motivated a systematic inquiry into the estimation of ionic conductivity, and study of ion transport in the KOH and EG mixture. Ionic conductivity also plays an important role in determining the energy efficiency of electrolytic and galvanic cells used in manufacturing, energy storage,^12,13^ surface treatment,^14^ and separation processes.^15,16^ Owing to the wide range of applications, the electrolytes need to be tailored to specific physicochemical property requirements.^17−20^ Appropriate choice of ionic and molecular constituents can meet these specifications; however, due to an extremely large chemical space of ionic and molecular species, it is impractical to evaluate all formulations experimentally. Computational techniques^21−23^ facilitate in-silico property estimation and high-throughput screening^24^ to identify relevant formulations, thereby accelerating the electrolyte development process.^25^ Furthermore, computational techniques can provide unique spatial and temporal insights into microscopic structure^26,27^ and molecular behavior that can complement experiments. It is important to note that the accuracy of these methods is sensitive to simulation parameters, including force fields,^28−30^ and errors in simulation parameters often translate to substantial offsets between the predicted and experimental values. Hence, it is important to assess the impact of force field and simulation parameters before deploying molecular simulations to predict ionic conductivity or deducing molecular insights about charge transport.

Several force fields have been developed to model behavior of alkali ions in aqueous environments such as those containing proteins, or across lipid bilayer membranes, since such environments are common in biological systems.^31−36^ These force fields have been developed for specific applications, and the parameters and combination rules were optimized to suit particular types of systems. In this work, we compare the performance of four commonly used force fields to represent alkali ions: (1) AMBER-99^37,38^ force field implemented widely to model systems such as nucleic acids; (2) CHARMM force field and its extensions that offer capability to model complex molecules and their interactions with a large number of solvents;^39,40^ (3) Jensen force field^41^ that was optimized for modeling halide, ammonium, and alkali metal ions in aqueous systems; (4) and also the all-atom optimized potentials for liquid simulations (OPLS)^42^ force field that was developed to match thermodynamic properties, gas-phase structures, and conformational energetics derived from ab initio calculations to describe accurately a wide range of fluids. These four force fields are selected for comparison due to their broader modeling capability and their ability to capture trends in physicochemical behavior of a wide variety of systems. Also, due to their compatibility with other force fields, they widen the chemical space to mixtures of solvents. Typically, in situations that demand high accuracy, ab initio techniques have also been used because these techniques do not rely on the accuracy of force fields to predict properties. For example, with a growing interest in finding cheaper, more abundant alternatives for Li^+^, Na^+^ and K^+^ based electrochemical energy storage devices are gaining interest.^43,44^ Since these systems are typically nonaqueous, ab initio methods have been used to predict the properties of these ions in nonaqueous solvents.^45−50^ However, first-principles techniques are system-specific, slow, resource-intensive, and thus impractical for situations that demand high-throughput screening. Due to the wide range of nonaqueous solvents, it is challenging to develop a generic force field that can represent all systems adequately. Hence, the scope of this work is limited to evaluating the usability of the aforementioned classical force fields to predict the ionic conductivity in nonaqueous electrolyte.

First part of this work estimates ionic conductivity of KOH in EG using four force fields used to represent the K^+^ ions and compares these estimates with the experimental measurements. Next, a thorough investigation into the molecular organization of the electrolyte is carried out to obtain an insight into the degree of dissociation, ion aggregation, charge distribution, and cluster dynamics. These analyses also help to rule out any unphysical behavior in the systems. Finally, we identify a force field that predicts Einstein conductivity values within 20–25% of experimental measurement, while adequately representing the underlying system.

## Methods

### Experiment

#### Materials and Sample Preparation

EG (99% purity) and KOH (99.99% purity) pellets were obtained from Sigma-Aldrich. EG was dried in a vacuum oven at 60 °C for 12 h prior to use in an experiment. Samples of concentrations in the range 0.25 to 1.0 mol/kg KOH were prepared by dissolving appropriate amounts of KOH in EG using a magnetic stirrer at 250 rpm for 3 h.

#### Density and Conductivity Measurements

The ionic conductivity of the samples was measured using a conductivity meter (Orion Star A212 Conductivity Benchtop Meter, Thermo Scientific, USA) and a conductivity probe (Orion 013005MD, Thermo Scientific, USA) at ambient pressure. All the measurements were conducted at temperatures between 298 and 333 K, with an accuracy of 0.05 K, and controlled by a thermostat (PT100 Probe, Chemglass Life Sciences, USA). The density of the samples was estimated gravimetrically by weighing samples of known volume on an analytical balance (Accuris W3100-210) with an accuracy of 0.0001 g.

### Simulation

#### Force Fields

Molecular interactions for EG were represented using the force field developed by Doherty and Acevedo,^51^ derived from the nonpolarizable OPLS-AA^42^ all-atom force field. This force field represents the intramolecular bonded interactions using harmonic stretching, bond angles, and dihedrals, while the long-range nonbonded interactions are represented using Coulombic and 12–6 Lennard-Jones (LJ) terms. The total energy is estimated using the following equations
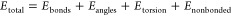
1
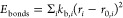
2
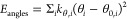
3
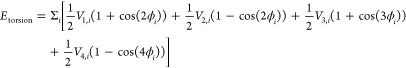
4
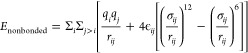
5where *k*_*b*_ and *k*_θ_ are the force constants for bond stretching and angle bending, respectively, and *V*_*i*_ denotes the Fourier coefficients for torsion in dihedral interactions. σ and ϵ represent the size and energy parameters for the 12–6 LJ interactions, while the electrostatic charges are given by *q*. LJ parameters for unlike interactions were estimated using geometric combination rules where  and . Both LJ and Coulombic 1–4 nonbonded interactions were scaled using a factor of 0.5. A cutoff distance of 16 Å was used to apply the tail corrections for LJ and electrostatic interactions, while the PME method was used to compute the long-range part of the electrostatic energy.

The hydroxide ion OH^–^ was modeled using a two-site nonpolarizable force field with a net charge of −0.75 developed by Habibi et al.,^52^ and the OH bond length was constrained to 0.98 Å. Four different force fields were used to represent the K^+^ ion by using the σ and ϵ values from AMBER,^53^ CHARMM,^40^ Jensen,^41^ and OPLS^42^ force fields, respectively. These force fields are referred to as FF1, FF2, FF3, and FF4, respectively. A net charge of +0.75 was assigned to match the charge on the OH^–^ force field. The ϵ values for FF1, FF2, and FF3 are similar and smaller by a factor of at least 170 compared to that for FF2. The extremely small magnitude of ϵ is also an indication that the interactions are predominantly repulsive. Considering the magnitude of σ, among the three force fields, the strength of repulsion follows the order FF1 < FF4 < FF3. The force field parameters are listed in Table 1.

**Table 1:** Force Field Parameters for K^+^ and OH^–^ Employed in This Study

force field	σ (nm)	ϵ (kJ/mol)	charge (e)
K^+^			
FF1 (AMBER)^53^	0.474	0.0014	+0.75
FF2 (CHARMM)^40^	0.314	0.364	+0.75
FF3 (Jensen)^41^	0.517	0.0021	+0.75
FF4 (OPLS)^42^	0.4934	0.00137	+0.75
OH^–^			
O	0.3650	0.251	–1.2181
H	0.1443	0.184	0.4681

#### Simulation Details

All molecular dynamics (MD) simulations were carried out using GROMACS 2018 package.^54^ To test the performance of the force field used to model the EG density, a system of 1024 EG molecules was simulated at different temperatures. The predicted density values were compared with the reported experimental values. Next, 30, 60, 90, and 120 ion pairs of KOH in combination with 2184 molecules of EG were used to represent approximately 0.25, 0.50, 0.75, and 1.0 mol/kg KOH in EG solution, respectively. Temperature effects were studied by simulating the systems at five temperatures: 298, 303, 313, 323, and 333 K at 1.0 bar.

The simulation protocol involved multiple steps. An initial configuration was generated using PACKMOL,^55^ by randomly packing the molecules into a cubic simulation box of initial volume estimated assuming ideal mixing, followed by a steepest descent energy minimization step to eliminate any high-energy configurations. To facilitate system relaxation, a high-temperature annealing scheme was implemented for 2 ns during which the system temperature was raised from 0 K to the desired temperature linearly and then maintained at the required temperature for about 300 ps before raising it further additional 200 K higher than the required temperature. The system was then maintained at the higher temperature for approximately 300 ps, cooled linearly, and maintained at the required temperature. The annealed system was then simulated using a canonical *NVT* ensemble followed by an isothermal–isobaric *NPT* equilibration for 10 ns each. Velocity-rescale thermostat^56^ and Berendsen barostat^57^ were used to maintain the temperature and pressure during equilibration steps, with coupling time constant of τ_T_ = 2.0 ps and τ_P_ = 4.0 ps, respectively. An additional 10 ns preproduction run was carried out to allow further equilibration. Finally, a 50 ns production run was carried out using a time step of 1 fs, and the coordinates were recorded at a 1 ps interval. Nosé–Hoover thermostat^58^ and Parrinello–Rahman barostat^59^ were used during the preproduction and production runs with coupling time constants τ_T_ = 2.0 ps and τ_P_ = 10.0 ps, respectively. The equations of motion were integrated using the leapfrog integrator. The trajectory thus obtained was used for the estimation of ionic conductivity and to carry out a molecular level analysis. The same initial configuration was tested for all four force fields by using the protocol discussed above. Simulations were carried out by using three different initial packing configurations to obtain statistical uncertainties.

### Ionic Conductivity Estimation

Ionic conductivity is commonly estimated using various approaches such as Nernst–Einstein,^60,61^ Einstein,^62^ or Green–Kubo^63^ method. These approaches differ from each other in the way they treat the interactions between the individual charge carriers. The Nernst–Einstein approach assumes that there are no interactions between charge carriers and uses the self-diffusion coefficients to estimate the ionic conductivity. The neglect of the ionic interactions implies that the ionic conductivity predictions are more reliable in the low-concentration regimes.^64−66^ In contrast, the Einstein formalism, although is rigorous in its treatment of ionic correlations, is slow and resource intensive and hence challenging to use for large simulation systems.^67^ Researchers have also explored hybrid approaches that model clusters of ions collectively as charge carriers, attempting to capture the essence of both techniques.^68^ Since the Nernst–Einstein and Einstein formalisms represent the extreme conditions, i.e., noninteracting and fully interacting charge carriers, these approaches were implemented in this work.

#### Nernst–Einstein Formalism

Mean square displacement (MSD) values for K^+^ and OH^–^ and the self-diffusion coefficients D_+_ and D_–_ were calculated from the production run trajectory where the MSD evolves linearly with time and used to compute the ionic conductivity of the system using the Nernst–Einstein equation. An ensemble average was calculated by averaging over different time origins and all the ions of a given type to efficiently capture the particle movements. Self-diffusion coefficients were calculated using the following equation
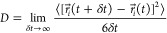
6where (*t*) is the position of the *i*th particle at time *t* and δ*t* is the time interval for which the magnitude of the displacement is calculated. The Nernst–Einstein equation was then used to predict ionic conductivity as a function of the concentration and diffusion coefficients of the ions. For a 1:1 salt, the Nernst–Einstein ionic conductivity is given by
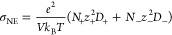
7where *k*_B_ is the Boltzmann constant, *e* is charge on an electron, *V* is the system volume, *T* is the temperature, and *N*, *z*, and *D* are the number of ions, charge, and diffusion coefficients of cations (+) and anions (−) respectively.

#### Einstein Formalism

Since the Einstein formalism captures the interactions between each particle of the system, it is calculated as a summation of interactions of every ion with all ions in the system. The following equation was used to estimate the overall ionic conductivity
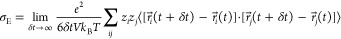
8where  and  are the positions of the *i*th and *j*th particles at time *t* and δ*t* is the time interval over which the dot product in the summation is calculated. The values were averaged over shifting time origins to reduce the statistical uncertainty of the Einstein conductivity estimates from the production run trajectory. A plot of the product term as a function of time was then constructed, and the slope of the curve during initial 1 ns where the dot product evolved linearly with time was used to estimate the Einstein conductivity, similar to that performed by Kapoor and Shah.^69^

## Results and Discussion

### Density

Reasonable estimation of the bulk density is an important factor in determining the accuracy of a force field. Since the electrolyte systems under consideration contained more than 94 mol % EG, first the performance of the OPLS_DES^51^ force field in predicting the density of pure EG at different temperature was assessed. Figure S1 illustrates the predicted densities compared with the experimental values. Although the force field consistently predicts densities that are lower than the experimentally reported values, the deviations were found to be within 1–2.5% with differences becoming greater with the temperature. Although the exact nature of increasing deviations from experimental values cannot be ascertained, possible reasons could be that the changing nature of hydrogen bonding with temperature is not accounted for; the change in polarity of the solvent may not have been captured by the force field. In fact, Doherty and Acevedo^51^ who developed the EG force field in the context of a deep eutectic system involving a salt, caution against using the force field for predicting properties of pure EG. However, since this work considers a mixture of EG and KOH, we accept these deviations to be within the margin of error, especially since not optimized to represent pure EG. Density values predicted by the four force fields for 1.0 mol/kg KOH solution were then compared with experimental values. Figure S2 represents the density of the electrolyte predicted at different temperatures. Density predictions are consistently lower for all of the force fields; however, the temperature dependence of the density is captured well by these force fields. Table 2 summarizes the mean absolute percentage error (MAPE) in the density predictions for 1.0 mol/kg KOH in EG, calculated using eq 9.
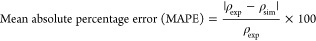
9where ρ_exp_ and ρ_sim_ are experimental and computationally estimated densities, respectively. The predicted densities lie within 3–4% of the experimental values for all of the force field models, suggesting the suitability of FF1, FF2, FF3, and FF4 models for the systems studied here. Although different force field models are used to describe K^+^ and OH^–^, all the force fields produce similar results as the density is primarily governed by the solvent force field, which as mentioned earlier, performs reasonably well in capturing the pure solvent density. Nonetheless, large deviations of the simulation results from experimental measurements for density can be used to eliminate a given force field. However, this was not the case here. Therefore, all of the force fields were considered for further evaluation.

**Table 2:** Mean Absolute Percentage Error in Density Estimates for 1.0 mol/kg KOH in EG Obtained Using Different Force Fields

	force field			
	FF1	FF2	FF3	FF4
MAPE (%)	3.78 ± 0.36	3.09 ± 0.37	4.22 ± 0.38	3.87 ± 0.38

### Ionic Conductivity

**Figure 1:**
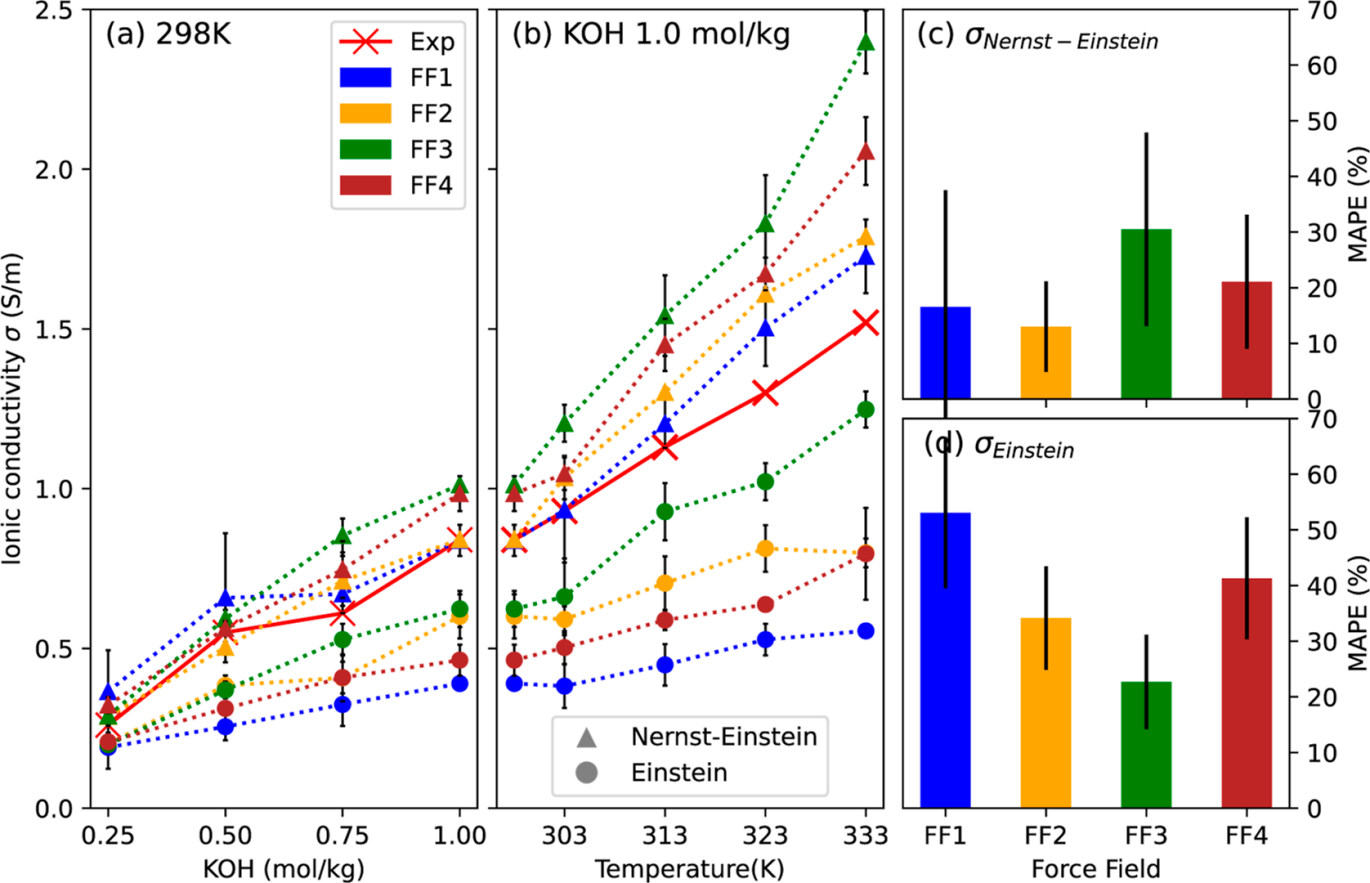
Ionic conductivity as a function of (a) KOH concentration and (b) temperature; MAPE in (c) Nernst–Einstein and (d) Einstein conductivity. Triangles represent the ionic conductivity calculated using the Nernst–Einstein approach, while circles denote those obtained from the Einstein formalism.

Figure 1a,b illustrate the predicted values of the ionic conductivity estimated using Nernst–Einstein (eq 6) and Einstein (eq 8) formalisms as a function of KOH concentration at 298 K, and as a function of temperature at 1 mol/kg concentration, respectively. Also included in these figures are the values of the ionic conductivity measured experimentally. Figure S3 shows examples of raw data for FF3 at 0.25 and 1.0 mol/kg KOH at 333 K that the Einstein term is linear for the duration over which the ionic conductivity is computed. It can be observed that the ionic conductivity increases with an increase in concentration of KOH, due to the increased number of charge carriers. Also, the ionic conductivity increases linearly with the rise in temperature, due to higher degree of dissociation and mobility of the charge carriers. All the force fields capture these trends reasonably well, within statistical uncertainty.

The Nernst–Einstein conductivity was generally higher, while the Einstein conductivity was lower than the experimental values for a given force field. These observations are consistent with the theory. Since the Nernst–Einstein equation ignores interactions among individual charge-carriers, it tends to estimate higher conductivity. On the other hand, Einstein conductivity estimates tend to be lower due to ion–ion correlations, and lower effective binary diffusion coefficients as compared to the self-diffusion coefficients estimated using Nernst–Einstein approach.

From Figure 1c, it can be observed that the Nernst–Einstein conductivity predictions obtained using FF1 and FF2 match closely with the experimental values, with the MAPE values between 10 and 20%. Lower MAPE and better resemblance with experimental measurements suggest the choice of FF2 or FF1 for estimating ionic conductivity. However, unlike the other three force fields whose accuracy worsened in the case of Einstein formalism, performance of FF3 improved, with an overall decrease in MAPE values to 20%. Better estimation of the Einstein conductivity highlights FF3′s advantages as evident from Figure 1d. It may be argued that KOH concentration of 1.0 mol/kg (5.5 mol %) or less may appear as relatively dilute and thus in the domain of Nernst–Einstein formalism. However, it was observed experimentally that the KOH concentration could not be increased much further without retention of insoluble KOH residues in the solution. This suggested that 1.0 mol/kg concentration was closer to the solubility limit of KOH in EG, and hence, assumptions of uncorrelated Nernst–Einstein ion transport were likely violated. This was further evident from the ion-agglomeration and cluster analysis discussed later in this work. Hence, the use of the Einstein formalism was deemed more appropriate, and FF3 is preferred for the calculation of ionic conductivity.

To provide a perspective on the effect of solvent, the ionic conductivity of 1 mol/kg of aqueous KOH at 298 K is measured to be about 19.8 S/m, while that for a similar solution in ethylene glycol is about 0.85 S/m. Water, due to its higher dielectric constant and polarity, is able to dissolve KOH with a much higher degree of dissociation. Furthermore, the water molecules shield the ions from each other, weakening the ion–ion interactions and inhibiting ion aggregation, thereby enhancing the ionic conductivity multifold, as compared to the less polar ethylene glycol. Hence, systematic inquiry into understanding the molecular level interactions is essential for the development of better and more efficient solvents.

### Self-Diffusion Coefficients

The self-diffusion coefficients for K^+^ and OH^–^ were calculated using eq 7 applied on the portion of the trajectory where the mean square displacement of ions evolved linearly with time, some examples of which are provided in Figure S4. Figure S5 in the SI illustrates trends in self-diffusion coefficients as a function of KOH concentration and temperature. It is noteworthy that the self-diffusion coefficients estimated by all of the force fields are higher for K^+^ than those of OH^–^, despite the higher mass of the cation. This observation indicates that the size of the diffusing species exerts more control on the diffusion of the species. Figure S5a indicates that the force fields FF1, FF2, and FF4 predict an inverse relation between the self-diffusion coefficient values and KOH concentration, which is expected as the increase in the number of charge carriers also increases the ion–ion interactions that may lead to formation of ion-pairs or aggregates, all of which hinder the ion diffusion. Self-diffusion coefficients computed using FF3 follow rather a complex trend in which the self-diffusion coefficients of K^+^ increase linearly with the concentration up to 0.75 mol/kg before showing a drop at 1.0 mol/kg. In contrast, the self-diffusion coefficients of OH^–^ exhibit a slight increase in going from 0.25 to 0.5 mol/kg. At low concentrations, the self-diffusion coefficients estimated by FF1 exceeded those predicted from other force fields; however, FF1 actually exhibits the lowest self-diffusion coefficients at the higher KOH concentrations. This behavior most likely originates due to the formation of ion clusters in FF1 at higher concentrations, slowing down the ion diffusion. It to be noted that although the simulations were carried out in an *NPT* ensemble in which the transport properties could be affected by the wrapping algorithm,^70^ given the large system size, it is expected that these effects would be negligible. In addition, the time constant for the thermostat could influence the transport properties. In the Supporting Information (Figure S6), it is shown that these effects are negligible, and the overall trend of ionic conductivities is preserved.

Table 3 compares the self-diffusion coefficients for dilute solution of K^+^ and OH^–^ in water at 298 K determined experimentally,^71^ through simulation,^52^ and that in ethylene glycol (at 0.25 mol/kg) as estimated in this work. The motivation is to understand the contribution of the solvent on ion-diffusivity. The self-diffusion coefficients in water are several times higher than those in EG, which is expected due to increased polarity, smaller size, and higher dielectric constant of water when compared with EG. The self-diffusion coefficients for K^+^ estimated by Habibi et al.^52^ in water match closely with the experimental values. However, the diffusivity of OH^–^ in water is underestimated by approximately a factor of 4. There are a couple of reasons for this observation: the self-diffusion coefficient of OH^–^ in water was reported at 0.48 mol/kg in simulation, while the experimental value was measured at the infinite dilute concentration. The second, and probably more important, is that the mobility of OH^–^ is likely to be dramatically affected due to the proton transfer as discussed in detail by Tuckerman et al.^72,73^ The transfer of a proton along the hydrogen-bonded network of water through a Grotthus-type mechanism can also be viewed as the transfer of OH^–^ in the opposite direction. As classical simulations cannot capture this phenomena, the self-diffusion coefficients determined solely on the basis of the movement of OH^–^ will underestimate the self-diffusion coefficient measured experimentally. Despite the seemingly poor performance of the OH^–^ force field for aqueous systems, it is anticipated that such effects are likely to be negligible in a nonaqueous solvent such as EG.

**Table 3:** Self-Diffusion Coefficients for K^+^ and OH^–^ in Water and Ethylene Glycol at 298 K

	diffusion coefficient *D* (10^–9^ m^2^/s)		
species	water (experimental)^71^	water (simulation)^52^	ethylene glycol (this work at 0.25 mol/kg)
*D*_K_^+^	1.96	1.95	0.25–0.5
*D*_OH_^–^	5.27	1.23	0.22–0.24

### Radial Distribution Functions

Radial distribution functions (RDFs) were calculated to understand the solvation environment around the ions in an effort to understand how they may aid in the explanation of trends in the transport properties obtained with different force fields. RDF [*g*(*r*)] between the two species *i* and *j* is defined as the ratio of local density [ρ_*j*_(*r*)] of species *j* at a distance *r* from the reference species *i* to the bulk density of species *j* as given by eq 10
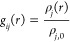
10

**Figure 2:**
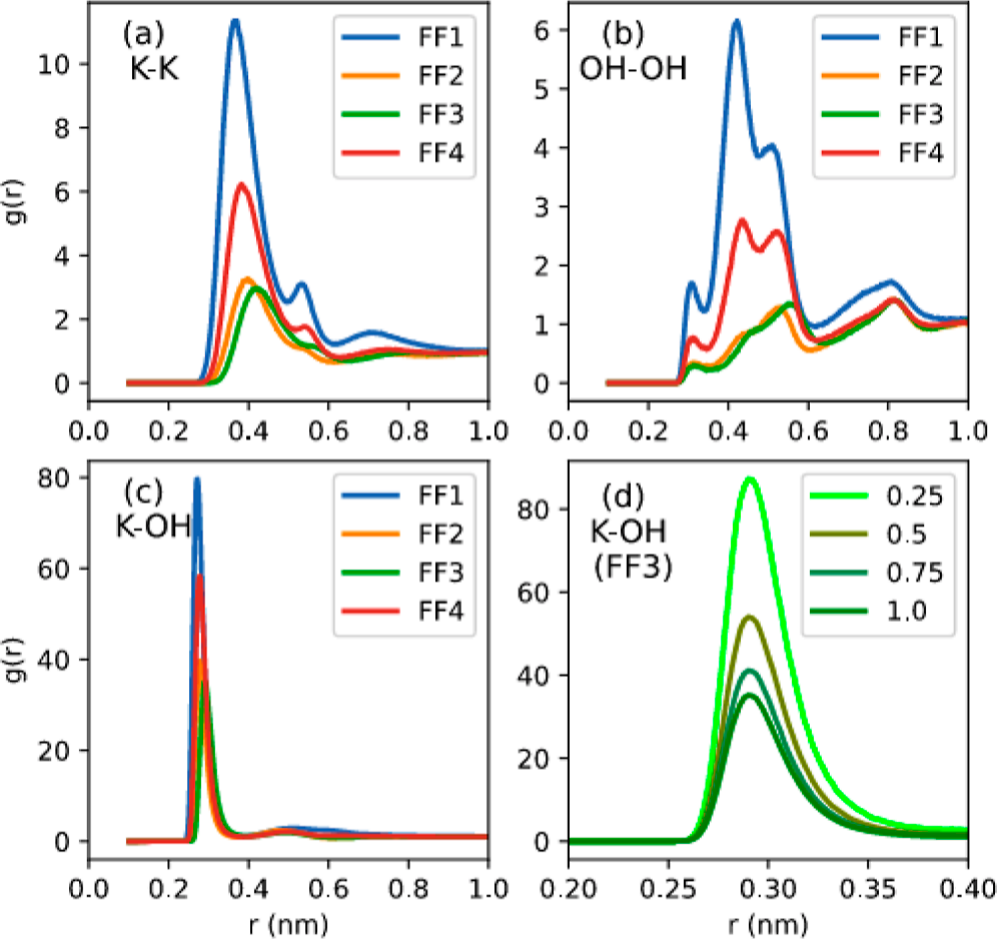
RDFs as a function of force field at 333 K and 1.0 mol/kg concentration for (a) K^+^–K^+^, (b) OH^–^–OH^–^, (c) K^+^–OH^–^, and (d) K^+^–OH^–^ at 333 K as a function of KOH concentration in mol/kg in EG.

Cation-centric (K–K, K–OH) and anion-centric (OH–OH) RDFs were generated using GROMACS and are provided in Figure 2 for different force fields. For OH^–^, the center-of-mass was considered for calculating the RDFs. Due to the relative masses of oxygen and hydrogen, the center-of-mass almost coincides with the oxygen position. Qualitatively, all of the force fields yield strong association between K^+^ and OH^–^ as oppositely charged ions aggregate due to strong electrostatic attraction. Repulsion between like charges prevents them from aggregating closely as in the case of opposite charges. This can be observed through the first peak heights in Figure 2, where the peak heights for K–OH RDFs are approximately one order of magnitude higher than those of K–K or OH–OH RDFs. Additionally, it can be observed that K–OH RDFs exhibit well-defined narrow first solvation shells (distance at which RDF attains the lowest value after the first peak). In contrast, the like-charge RDFs show expanded first solvation shells marked by multiple peaks. Although the nature of the peaks and maximum peak height vary significantly between the force fields, the radii corresponding to the size of the first solvation shell, for K–K and OH–OH RDFs, were found to be within a narrow range between 6.5 and 7.0 Å for all force fields. The peak heights follow the trend FF1 > FF4 > FF2 > FF3, while the distance of the first peak shows the opposite trend FF1 < FF4 < FF2 < FF3, indicating slightly stronger repulsion in FF3.

Delving deeper dive into K–K RDFs (Figure 2a), substantial differences exist for the various force fields. For example, in addition to the prominent first peak, the FF1 and FF4 force fields result in a shoulder before achieving a minimum, pointing to an aggregation behavior; such a shoulder is not as prominently visible in the RDFs calculated with either FF2 or FF3 force field. Additionally, the *g*(*r*) values for RDFs in FF1 stay above 1 up to 10 Å, providing additional evidence of aggregation when the FF1 force field is employed. The propensity to aggregate is enhanced as seen by increasing heights of all the peaks, as seen in Figure S7. In contrast, *g*(*r*) values approach 1 immediately following the first shell minima for the other three force fields. The observation also implies that the structuring of K^+^ around itself is limited only to the first solvation shell when either FF2, FF3, or FF4 force field is employed. The RDFs also appear to be insensitive to changes in the concentrations (Figure S7c,d) for FF2 and FF3 force fields.

Similar to the K–K RDFs, the anion–anion RDFs in Figure 2b illustrate significant differences in the nature of the peaks observed for the various force fields in the first solvations shell. A key difference is that the first solvation shell is marked by three peaks for FF1 and FF4, all of which display intensity above unity suggesting the enhancement in the density of the anion over the bulk density. The distance between successive peaks is approximately 1 Å, which is equivalent to the OH bond length. In sharp contrast, the *g*(*r*) values below unity, in the RDFs for FF2 and FF3 force fields, imply that there is a depletion in the anion density in the first solvation shell in comparison to the bulk density. An examination of the concentration dependence of anion–anion RDFs in Figure S8 reveals a more pronounced effect of the KOH concentration on the peak heights in FF1. The other force fields do not exhibit such deviations in *g*(*r*) as a function of the bulk KOH concentration.

The cation–anion RDFs in Figure 2d depict the dependence of local density on the bulk KOH concentration. Although the results are provided only for the FF3 force field, Figure S9 shows similar trends across all force fields. The peak heights are highest for the 0.25 mol/kg KOH solution and then drop monotonically with increasing KOH concentration. The decrease in height can be explained by considering that only a limited number of ions of opposite charge can be accommodated in the first solvation due to the excluded volume effect and repulsion between the counterions. This effect can be easily seen in the coordination number plot in Figure S10, which demonstrates that the number of OH^–^ ions in the first solvation shell around K^+^, although increasing linearly, does not scale in proportion to the bulk density, which results in the drop in the first peak height. An examination of the coordination numbers from Figure S10b also supports this and reflects a higher degree of ion agglomeration in FF1. RDFs thus indicate presence of ion-aggregates, but lack specific details about the size, charge, etc. of these ion aggregates. This necessitates further inquiry into the cluster composition and charge dynamics of these ion-aggregates.

### Ion-Aggregation and Cluster Analysis

**Figure 3:**
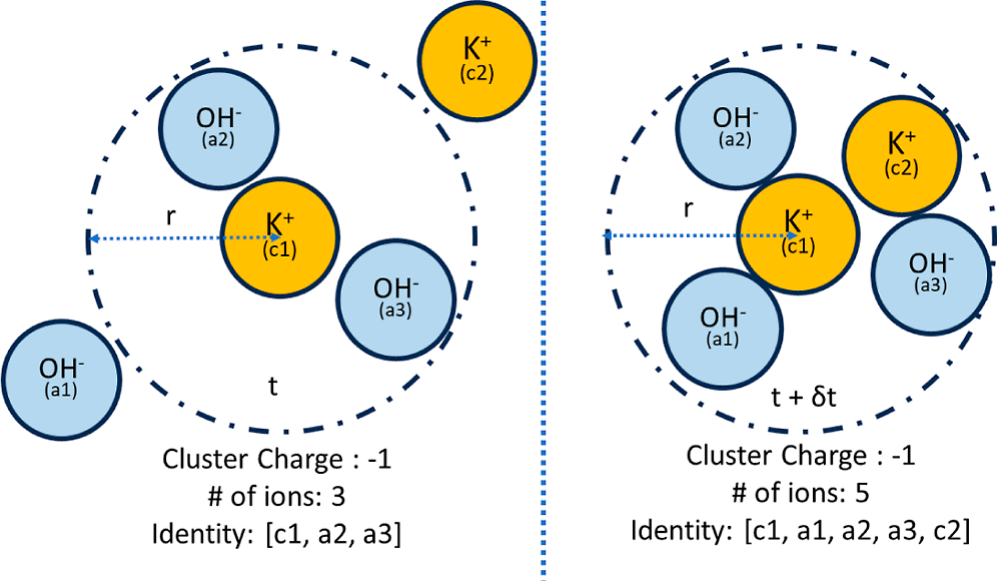
Defining cluster size, charge, and identity. Note that at time *t* and *t* + δ*t*, the overall charge of the cluster is −1; however, the cluster identity has changed due to the migration of ions a1 and c2 into the coordination sphere of the central ion (c1).

Ion-aggregation violates the assumptions of the Nernst–Einstein approach due to the strong ion–ion interactions, thereby causing deviation from the expected ionic conductivity values. To examine the aggregation behavior, several analyses based on identifying ionic clusters were carried out. A cluster is defined as a spherical region of radius *r* around a reference ion as shown in Figure 3a. As RDFs indicate the radii of solvation shells for like-charge ions to be between 6.5 and 7.0 Å, a radius of 6.8 Å was selected for cluster analysis. At higher KOH concentrations, all force fields indicate some tendency toward ion-aggregation. However, FF1 predicted the strongest aggregation among all of the force fields, followed by FF4. Approximately similar solvation environments were obtained with FF2 and FF3. Analyses from FF1 and FF3 are presented in the following sections since these force fields represent the extremes in estimation of ionic conductivity via the Nernst–Einstein and Einstein approaches. Analyses from FF1 and FF3 are presented in the following sections since these force fields represent the extremes in estimation of ionic conductivity via the Nernst–Einstein and Einstein approaches (Figure 1d).

#### Ion Count Distribution

**Figure 4:**
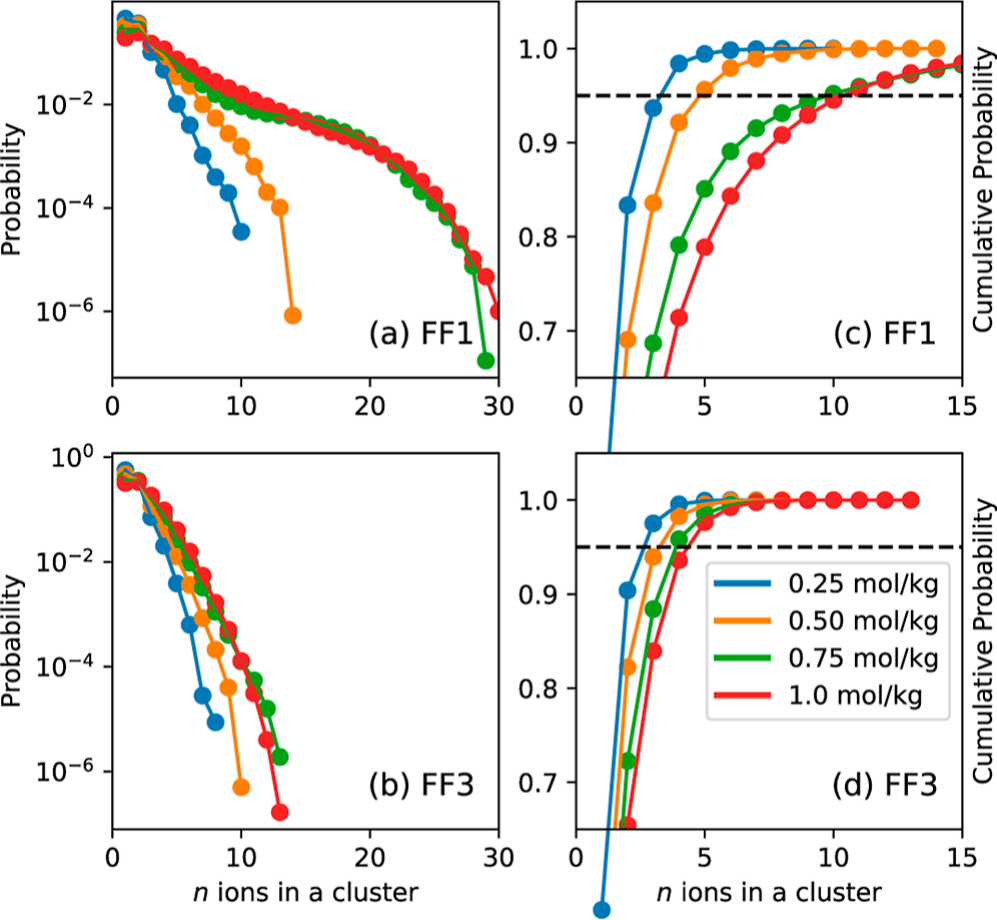
Average and cumulative probabilities of finding clusters containing total of *n* ions as a function of KOH concentration at 313 K in FF1 (a,c) and FF3 (b,d). Black dotted line represents 95% cumulative probability.

As a first step to study ion-aggregation, the local environment around each reference ion was probed to obtain the total number, type (cation/anion), and distance of each ion within a cluster of radius 6.8 Å. Figure 4a,b illustrates the average probability of finding a cluster containing a total of *n* ions for FF1 and FF3, respectively. It is to be noted that, by definition, a cluster can contain both cations and anions, and no distinction is made regarding the identity of the central ion. It can be observed that the probabilities drop off rapidly with an increasing number of ions. Hence, for better visualization, they are shown on a logarithmic scale. In addition, it is also evident that the probabilities of finding a given number of ions increase with an increase in the KOH concentration from 0.25 to 0.75 mol/kg; the probabilities appear to be nearly indistinguishable for KOH concentrations of 0.75 and 1.0 mol/kg. A strong tendency toward aggregation is seen while using FF1: the maximum number of ions in a given cluster is found to be close to 30 at the highest concentration. In the case of FF3, the maximum number of ions is about 15. At concentrations studied in this work, large agglomerates such as these are expected to be rare and transient, hence, the cumulative distributions were also examined and are provided in Figure 4c,d for the two force fields. At higher concentrations, 95% of clusters for FF3 contain a total of five ions or less, while this number is nearly double at 10 or fewer ions for FF1. Figure S11 illustrates how the total number of ions in a cluster varies as a function of time for all the force fields in 1.0 mol/kg KOH solution.

#### Ion Proximity Distribution

**Figure 5:**
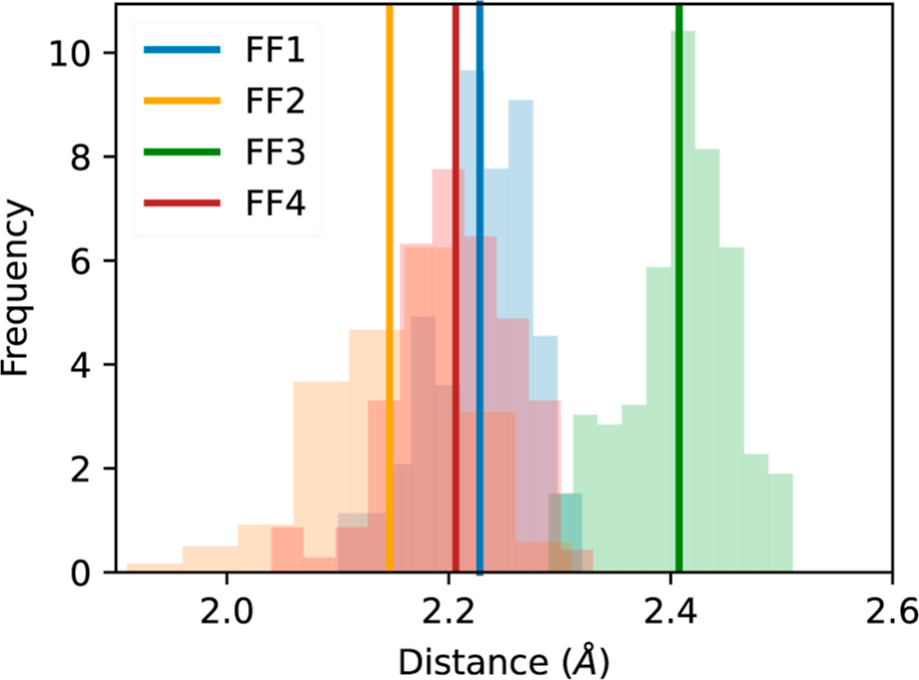
Distribution of shortest distances within a cluster at 1.0 mol/kg and 333 K for different force fields.

The presence of as many as 10 or more ions within 6.8 Å around the reference ion for the FF1 force field requires that a proximity analysis be carried out to eliminate the possibility of ion-overlap. Ions can approach close to each other due to high electrostatic attraction and/or solvophobic effect; however, the repulsive LJ interactions maintain the physicality of the system by preventing overlap. If the LJ repulsive interactions are weaker than the electrostatic attraction, it can lead to an overlap or the ions getting unphysically closer to each other. The proximity of ions was assessed by recording the distribution of the shortest interionic distances within each cluster. Figure 5 depicts these distributions in 1.0 mol/kg KOH systems at 333 K. The solid lines indicate the mean shortest interionic distance for a given force field, which are between 2.15 and 2.4 Å with the shortest distance obtained with FF2 force field and the largest with FF3 force field. This indicates that the LJ parameters of FF3 exert stronger repulsive interactions in comparison to those experienced in other force fields.

#### Cluster Charge Distribution

**Figure 6:**
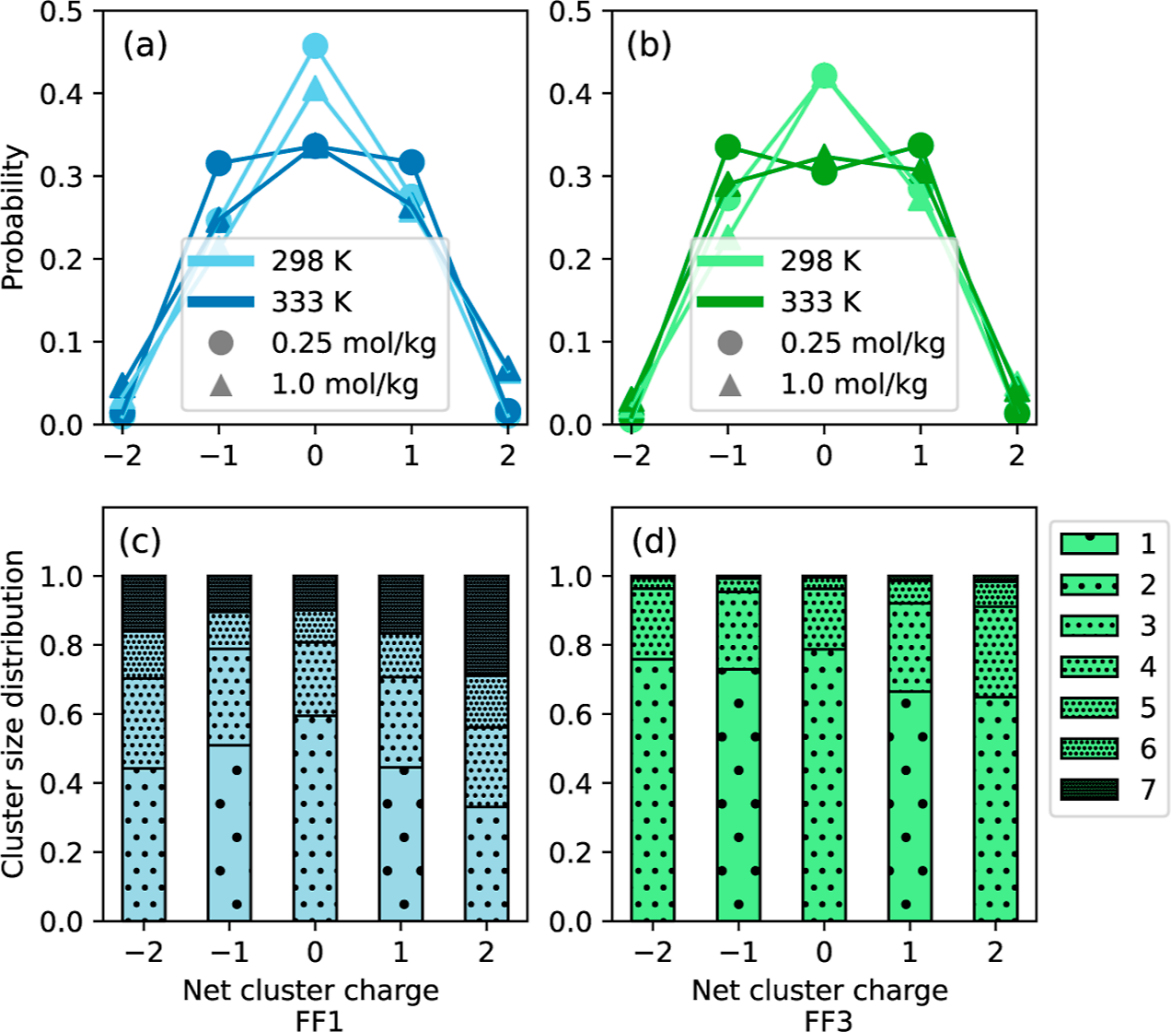
Probability distribution of net cluster charge at 298 and 333 K in 0.25 mol/kg and 1.0 mol/kg solutions, predicted by (a) FF1 and (b) FF3; ion-composition of cluster as a function of net charge in 1.0 mol/kg solution at 333 K predicted by (c) FF1 and (d) FF3. The hatching patterns reflect the total number of ions that form the cluster. All clusters containing more than 7 or more ions are clubbed together.

Analysis of the ionic composition of a cluster is essential for determining the net charge carried by the cluster, which can inform the types and relative populations of the clusters available for charge transport. For example, when the numbers of cations and anions are equal, the cluster is charge neutral and does not contribute to ionic conductivity. On the other hand, any surplus in cations or anions yield a cluster carrying a net positive or negative charge respectively, contributing toward ionic conductivity. Figure S12 depicts the evolution of cluster charge during the production run for all the force fields in 1.0 mol/kg KOH solution at 333 K. Figure 6a,b represents the average probability of finding a cluster with a given net charge around a randomly selected reference ion from the system obtained with FF1 and FF3 force fields, respectively. Also shown in these figures are the effects of the concentration and temperature on the cluster charge distributions. Results from both the force fields indicate that the overall charge carried by the clusters in these systems varies between −2 and 2 with significant probabilities for charges −1, 0, and +1. In fact, about 40–45% of the clusters are charge neutral at 0.25 mol/kg, which lowers the Einstein conductivity in comparison to the Nernst–Einstein conductivity. At a higher temperature of 333 K, the fraction of the charge neutral clusters drops to approximately 30%, thereby increasing the ionic conductivity. Both FF1 and FF3 predict similar behavior that is consistent with the expectation of higher degree of dissociation due to an increase in temperature.

Figure 6c,d shows the cluster size distribution as a function of net cluster charge in a 1.0 mol/kg solution at 333 K. The hatching patterns depict the total number of ions present in a cluster. The ionic composition can also be deduced from this information; for example, clusters carrying a +1 charge with 5 ions will contain 3 cations and 2 anions, etc. It can be observed that even though FF1 and FF3 exhibit similar probabilities of finding clusters of a given charge, the ionic makeup of these clusters varies significantly. The cluster composition in FF3 is dominated (more than 70%) by dissociated ions or ion pairs. These numbers drop to between 40 and 50% in the case of FF1. When it comes to clusters containing 3 or 4 ions, the distribution is similar in both force fields. Five or more ions are uncommon in FF3, but these contribute to between 20 and 40% of the clusters with higher net charges in FF1. The Nernst–Einstein conductivity estimated by FF3 is nearly 1.4 times that of FF1, which may be expected based on relative ratios of dissociated ions or small clusters observed in these force fields.

### Spatial Analysis and Dynamics

Figure 3 illustrates how assigning unique tags to ions can be leveraged to monitor the identity of a cluster and its dynamics. Cluster identity is defined by combining the tags of all of the constituent ions. Any inflow or outflow from a cluster changes its identity. The net charge may or may not change, depending on the overall composition of the cluster. Figure S13 illustrates the relative number of unique identities in different force fields. It can be observed that the number of unique identities increases with both the temperature and KOH concentration. This may be attributed to faster diffusion of ions at higher temperature or due to the combinatorial effect in clusters containing a large number of ions, as in the case of higher concentrations. The increase in the number of unique identities from 298 to 333 K is about 20–30%, while that for 0.25 and 1.0 mol/kg at the same temperature is roughly 10-fold. In 0.25 mol/kg solutions, all force fields exhibit a similar number of unique identities per cluster; however, for 1.0 mol/kg solutions, FF1 exhibits nearly twice as many unique identities as other force fields, due to the ion aggregates forming in FF1.

**Figure 7:**
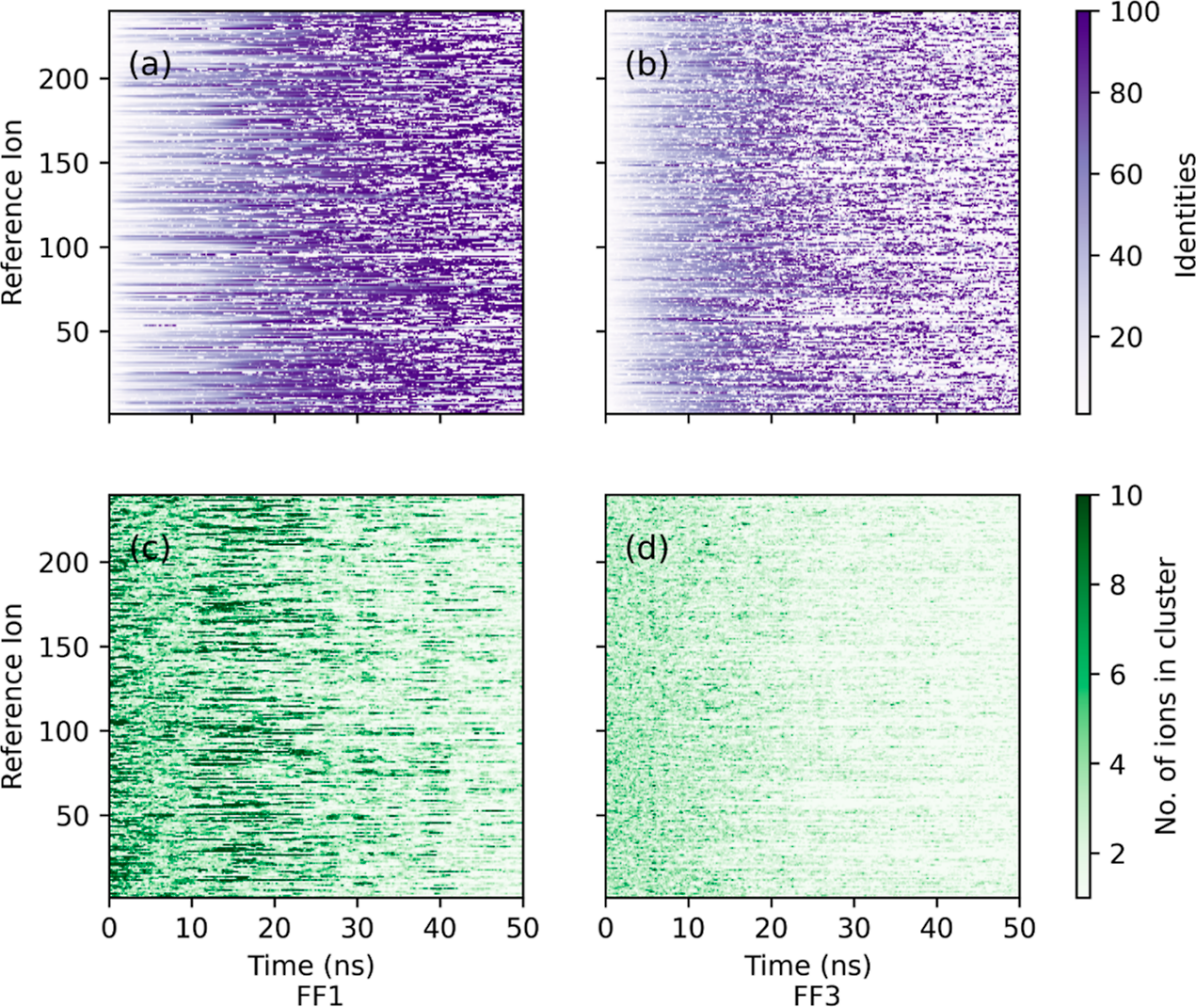
Cluster charge dynamics in 1.0 mol/kg KOH at 333 K over 50 ns trajectory predicted using FF1 (left pane) and FF3 (right pane). The top pane indicates how identities evolve over time while the bottom pane provides information on the number of ions in a cluster as a function of time.

To gain additional insights into how cluster identities evolve over time, identities were scaled to 100. Figure 7a,b depicts such dynamics for FF1 and FF3 force fields, respectively, for each of the ions in 1.0 mol/kg of KOH solution. The absence of a change in color indicates that the identity of the cluster surrounding a given ion has not changed. Such a behavior is exhibited by both the force fields for the first 5 ns or so. Over time the clusters transition to purple due to movement of ions. In case of FF1, the purple color is retained, indicating that the clusters tend to their final identity after they have undergone dissociation, indicating charge transport due to the diffusion of such clusters. On the other hand, several transitions between purple and white colors are observed, which point to ions hopping into and out of the clusters, leading one to conclude that the charge transport is not only due to the diffusion of cluster but also arises because of migration of individual ions. This observation is key to explaining higher ionic conductivity values obtained with FF3 force field. Figure S14 reveals how identities of clusters change over the entire production run for different force fields in 1.0 mol/kg KOH solution at 333 K.

#### Charge and Identity Correlations

Quantification of ion–ion correlations helps to determine the average time a given cluster maintains its charge and/or identity. For example, short correlation times indicate short-lived ion–ion interactions, while longer correlation times indicate the formation of ion-pairs or ion-aggregates. Correlated movement of oppositely charged ions is undesirable, as it does not contribute to net charge transport. On the other hand, correlated movement of like charges is favorable toward improving net ionic conductivity. Intermittent and continuous correlations were calculated to quantify such dynamics (eq 11).
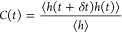
11where *h*(*t*) is the Heaviside function which assumes a value of unity at the time origin, while the value of *h*(*t* + δ*t*) is determined based on the type of the correlation function that is desired. For example, for the intermittent correlation function, *h*(*t* + δ*t*) is set to unity if the property of interest (charge or identity) considered is identical after a time interval of δ*t* or zero otherwise. On the other hand, the continuous correlation function requires that the quantity remains identical throughout the interval δ*t* for *h*(*t* + δ*t*) to be assigned a value of unity. The overall correlation function *C*(*t*) was calculated as an ensemble average at a given time step. Time averaging across different time origins was implemented to improve the statistical averaging. Cluster lifetimes were calculated as the area under the *C*(*t*) (eq 12).
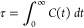
12

**Figure 8:**
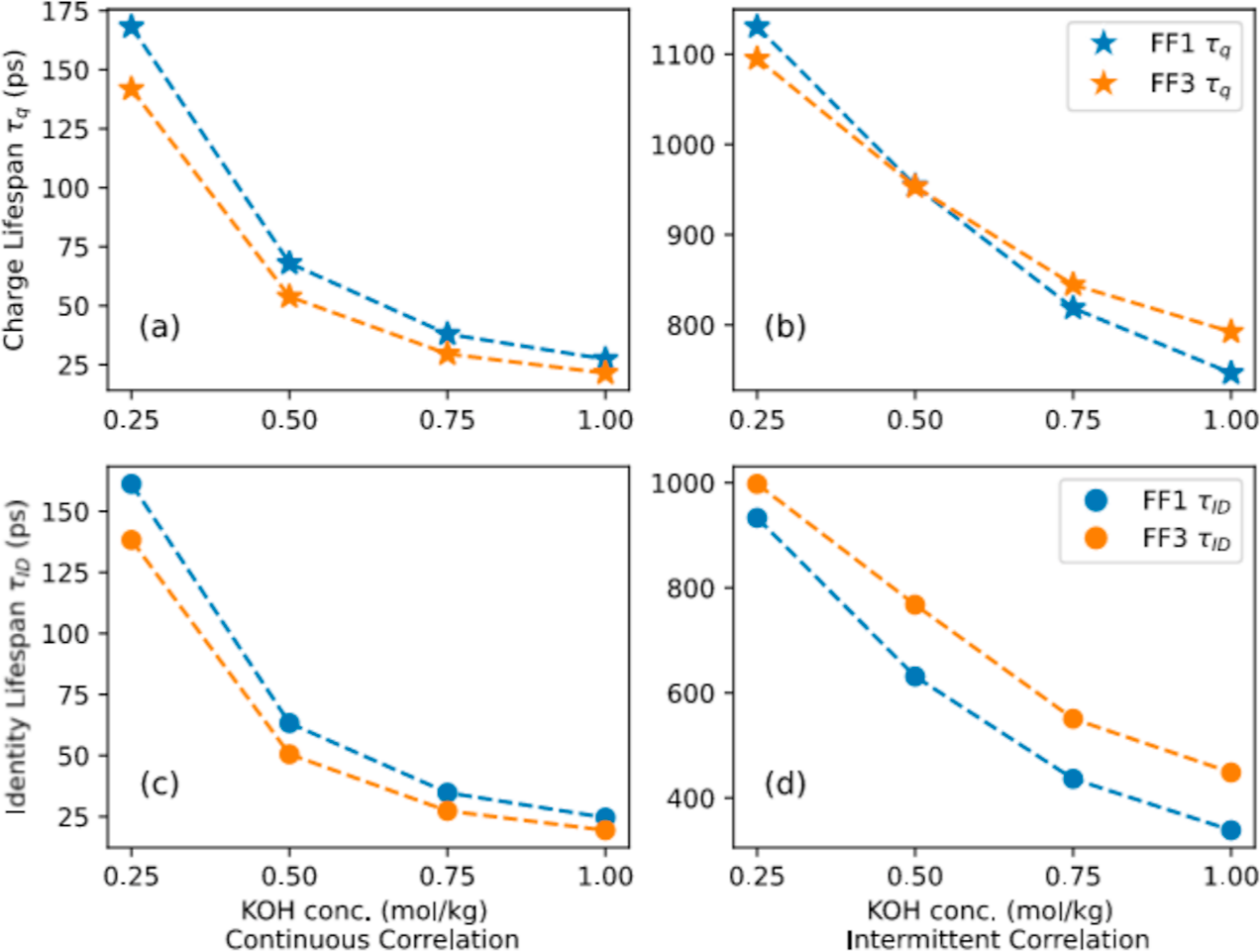
Cluster charge lifetimes continuous (a), intermittent (b) and cluster identity dynamics continuous (c) and intermittent (d) as a function of the KOH concentration at 313 K.

The continuous and intermittent cluster charge lifetimes (τ_q_) and cluster identity lifetimes (τ_ID_) for FF1 and FF3 are displayed in Figure 8. The corresponding correlation functions are included as the Supporting Information (Figure S15), which show that the charge and identity continuous correlation functions approach zero in roughly 2 ns for both the force fields; however, the intermittent correlation functions seem to plateau to a nonzero value with simulation time, indicating that there is a possibility of reformation of the original cluster even after a significant time has elapsed. Such behavior is qualitatively apparent from Figure 7. As a result, the continuous lifetimes are an order of magnitude lower than those obtained for the intermittent functions. Although lifetimes for intermittent correlation functions are provided by integrating the correlation function up to 2 ns, the actual values must be interpreted with caution.

From Figure 8a,c, it can be concluded that both cluster charge and identity correlation lifetimes decrease as a function of the concentration. At lower concentrations, infrequent encounters between clusters or dissociated ions are responsible for maintaining the cluster charge and identity for a longer period of time, while exchange of ions with other clusters become more probable at higher concentrations resulting in a faster change in cluster and charge identity. Cluster charge lifetimes for continuous correlations tend to be higher than corresponding identity correlation lifetimes, which can be understood based on the fact that it is easier to maintain the charge of a cluster simply by exchange of the same number and type of ions, but such an exchange alters the cluster identity. The continuous correlation function lifetimes are predicted to be higher for FF1 than those for FF3 due to a greater degree of dissociation observed in FF3 than that in FF1. Equivalently, the tendency for ions to aggregate is higher when FF1 force field is employed. The trend, however, was found to be opposite in the case of intermittent correlation functions. The greater degree of dissociation of ions in systems produced using FF3 force field leads to fewer ions in a cluster and their identities are simpler. These identities may reappear intermittently as ions move in and out of the clusters, thereby resulting in longer intermittent lifetimes.

Differences in ion-aggregation can be attributed to LJ parameters σ and ϵ. Among force fields with comparable well depths or similar ϵ values (FF1, FF3, and FF4), the size and lifespan of clusters are inversely related with the van der Waals radii, σ. FF1 with its shorter σ value promotes higher ion-aggregation, forming larger clusters. Larger clusters lead to stronger interionic correlations and consequently lower self-diffusion coefficients, Nernst–Einstein and Einstein conductivity values. On the other hand, FF3 with the larger σ value leads to a more dissociated system with low ion–ion correlations, thereby yielding higher conductivity values. The contrast between effects due to the LJ parameters becomes more pronounced at higher concentrations due to the higher overall number of ions and ion–ion interactions. We suspect the ion-aggregation observed in this work is similar to that described in a study^74^ by Chen and Pappu, although their study focused on monovalent ions in aqueous solutions of nucleic acids.

## Conclusions

This work focused on evaluating the ability of four commonly used force fields for K^+^ to perform ionic conductivity calculations of KOH in ethylene glycol as a function of concentrations. The Nernst–Einstein conductivity and Einstein conductivity values obtained from molecular dynamics simulations were compared with experimental values. The results indicated that out of the four force fields, labeled as FF1, FF2, FF3, and FF4, the FF3 force field yielded the overall best agreement with experimental ionic conductivity, while the deviations obtained from FF1 force field were the highest. The ionic conductivity trends were explained in terms of the propensity of ions to form aggregates that can be directly linked to σ and ϵ parameters of K^+^ in these force fields. For example, although values of ϵ for FF1, FF3, and FF4 were nearly identical, σ increases in the order FF1 < FF2 < FF3 which profoundly impacts the aggregation behavior: FF3 resulted in ion-agglomeration and large clusters and a slow vehicular mode of ion transport, while the repulsive nature of the FF3 force field promotes dissociation, thereby producing smaller, transient cluster and a diffusion-dominated charge transport. Enhanced aggregation is reflected in lower ionic conductivity values due to ion–ion correlation. Aggregate charge and identity dynamics also revealed that decorrelation times are higher for FF1 due to aggregation. Due to the propensity of these force fields for ion aggregation, caution must be exercised against benchmarking force fields solely on the basis of Nernst–Einstein conductivity. The analysis techniques implemented in this work also provide insights useful for understanding ion agglomeration and charge dynamics in similar electrolyte systems.

## Supporting Information Available

Comparison between experimental measurements and simulation predictions of ethylene glycol density and that between 1.0 mol/kg KOH in EG experimental densities and simulation predictions using various force fields, dependence of radial distribution function, cluster charge and identity, and number of unique identities as a function of various force fields. Raw data and sample simulation files are available for download from https://github.com/ShahResearchGroup/JPCB_EG_KOH (PDF)
