# Supplementary material for: Effect of K+ Force Fields on Ionic Conductivity and Charge Dynamics of KOH in Ethylene Glycol
Source: J Phys Chem B. 2024 Apr 4;128(15):3707–19. doi: 10.1021/acs.jpcb.3c08480 (PMC11033864; doi:10.1021/acs.jpcb.3c08480)
Supplement: Supplementary file 1 — jp3c08480_si_001.pdf [file jp3c08480_si_001.pdf]

Supporting Information: Effect of K^+ Force Fields on Ionic Conductivity and Charge Dynamics of KOH in Ethylene Glycol

Amey Thorat,† Rohit Chauhan,‡ Rohan Sartape,‡ Meenesh R. Singh,‡ and Jindal K. Shah*,†

†*School of Chemical Engineering, Oklahoma State University, Stillwater, OK - 74078, USA*

‡*Department of Chemical Engineering, University of Illinois at Chicago, Chicago, IL - 60608, USA*

E-mail: jindal.shah@okstate.edu

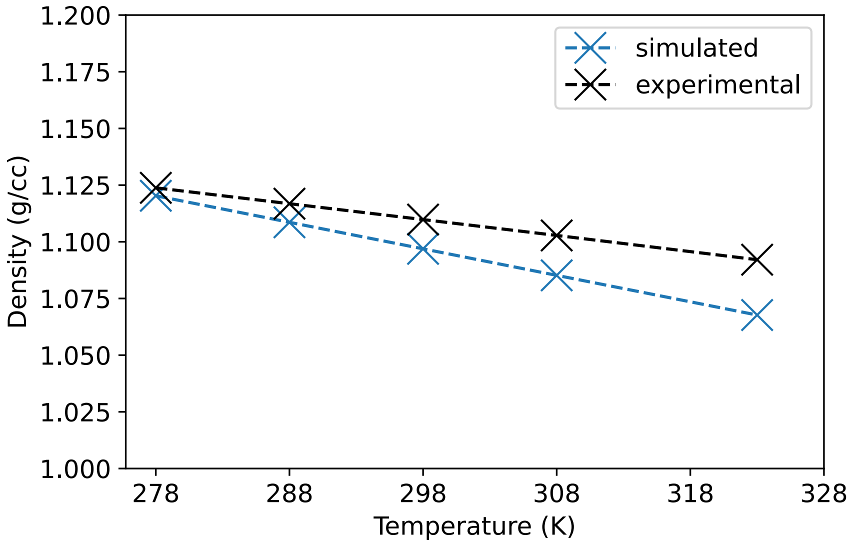

Figure S1: Density of pure ethylene glycol as a function of temperature

Ionic Conductivity using the Einstein Formalism

Figure S3 shows the evolution of the Einstein term $\sum_i \sum_j z_i z_j \Delta r_j \dot{\Delta}_j$ as a function of time. It can be observed that this term is linear for the first one ns, which is used to calculate the Einstein conductivity.

Self-Diffusion Coefficients

Self-diffusion coefficients were calculated based on the MSD values from the production run spanning 10 ns to 40 ns i.e. excluding the initial and final 10 ns of the trajectory. The following plots depict MSD vs t and values of non-Gaussian parameter $\beta(t)$, evaluated as the slope of log-log MSD versus time plot, which can also be expressed using the following equation:

$$\beta(t) = \frac{d \ln \Delta r^2}{d \ln t} \tag{1}$$

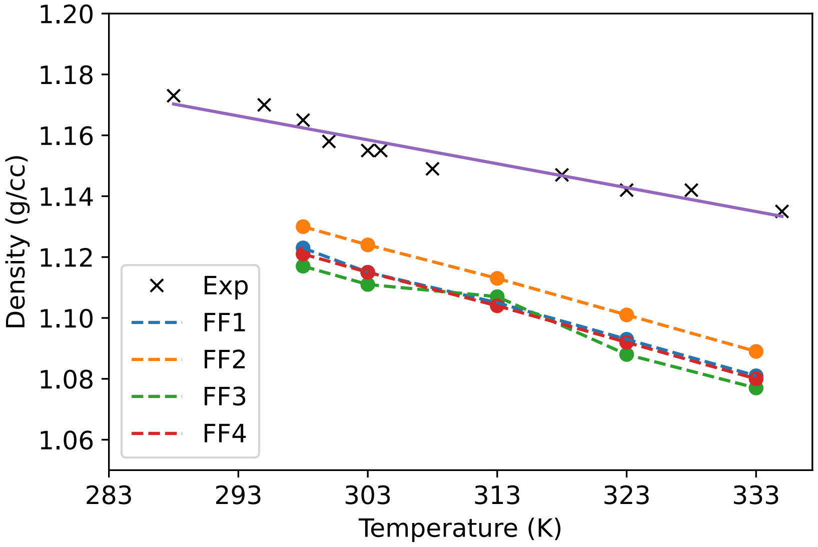

Figure S2: Density of 1.0 mol/kg KOH in ethylene glycol as a function of temperature

In diffusive regime the value of $\beta(t)$ approaches unity. This confirms that the systems are in the diffusion regime during the estimation of self-diffusion coefficients. To illustrate with an example, the following figures demonstrate the MSD and corresponding $\beta(t)$ values for K^+ and OH^+ in a 1.0 M KOH/kg and EG solution at 333 K modeled using FF3.

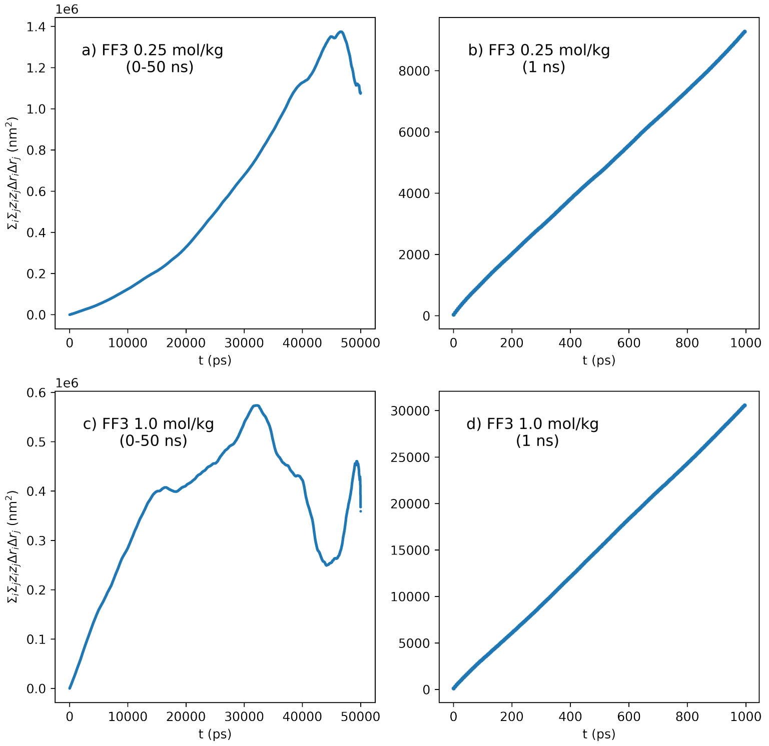

Figure S3: Einstein term for 0.25 mol/kg KOH (top pane) and 1.0 mol/kg KOH (bottom pane) over the entire simulation trajectory (left) and the first 1 ns (right) over which the Einstein conductivity is calculated. The data are shown for FF3 force field at 333 K.

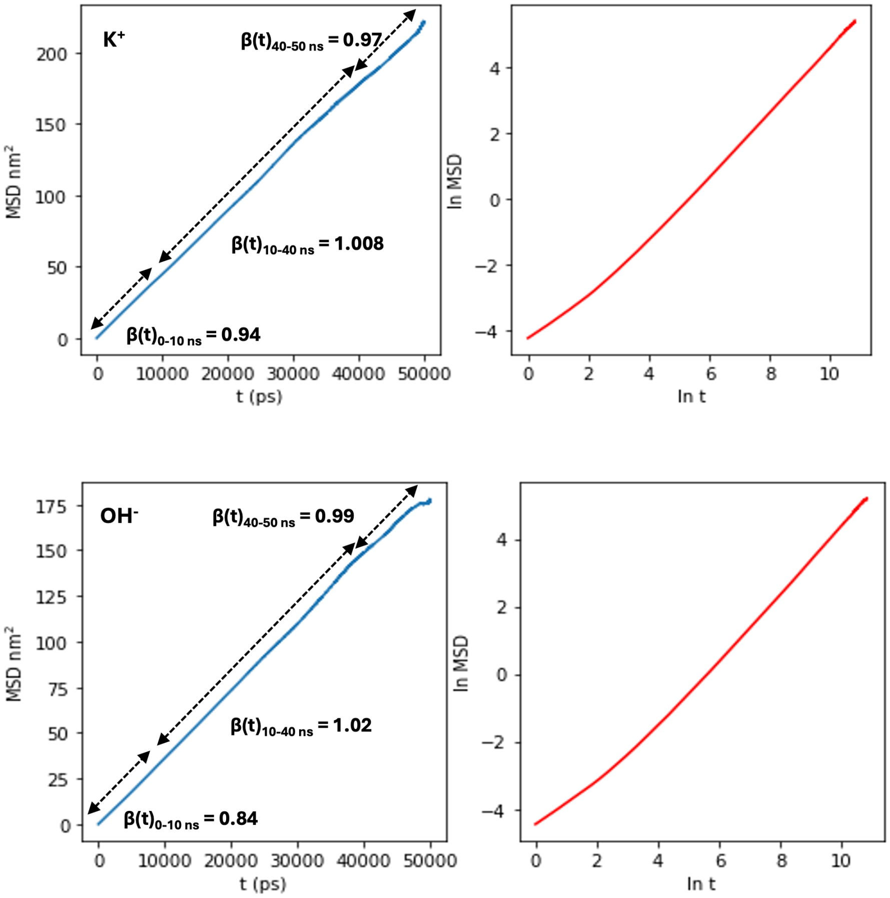

Figure S4: Mean-squared-displacement as a function of time and ln MSD vs. $\ln t$ for K^+ (top pane) and those for OH^- (bottom pane) for 1.0 mol/kg KOH in ethylene glycol at 333 K modeled using FF3 force field.

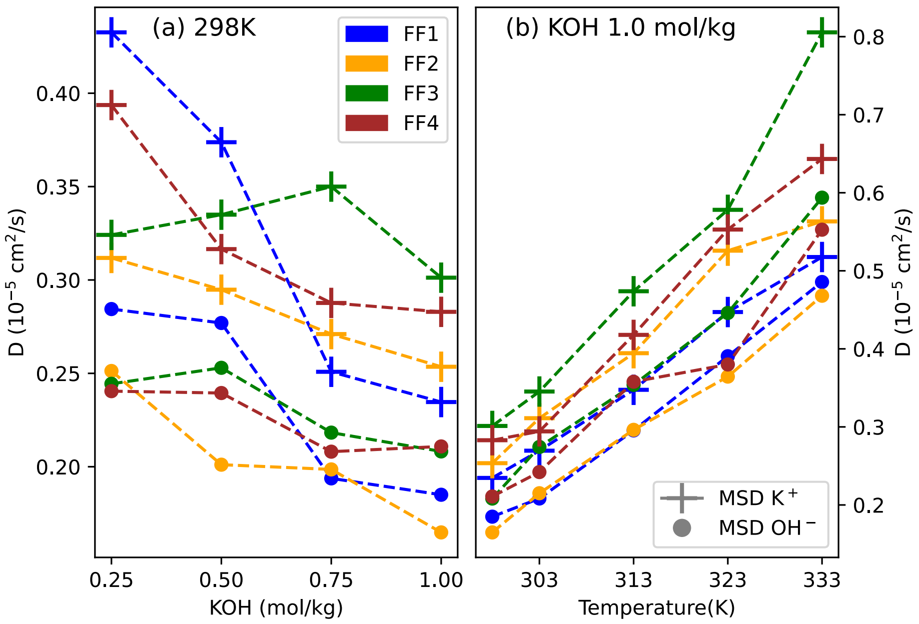

Figure S5: Self-diffusion coefficient of K^+ and OH^- ions as a function of a) KOH concentration (at 298K) and b) Temperature (at 1.0 mol/kg KOH)

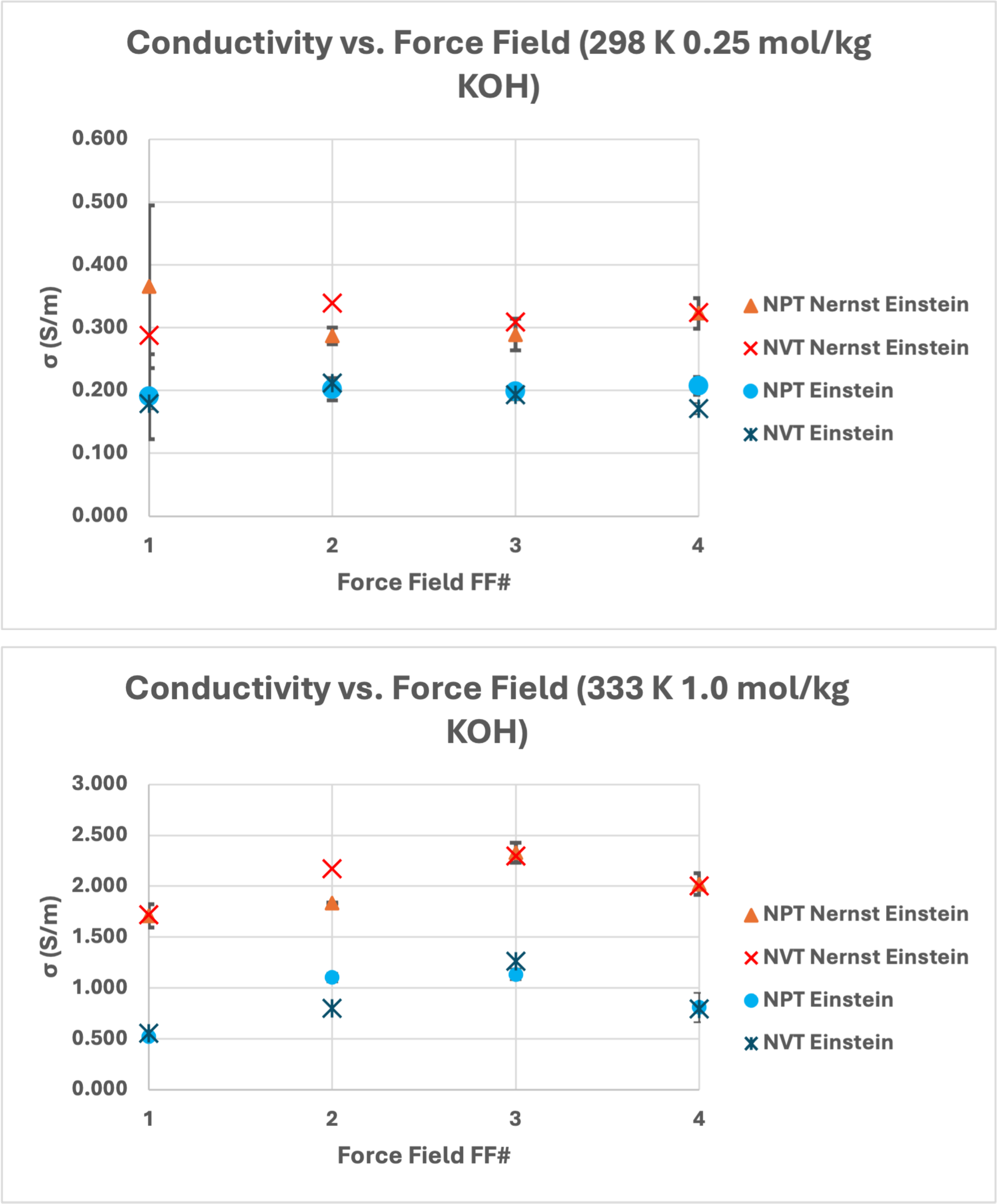

Figure S6: Einstein and Nernst-Einstein conductivity predictions using the NPT-protocol with $\tau_T = 2.0$ ps and $\tau_P = 4.0$ps and NVT simulations with $\tau_T = 0.4$ps. The top pane provides data for 0.25 mol/kg KOH while the bottom pan collects data for 1.0 mol/kg KOH in EG.

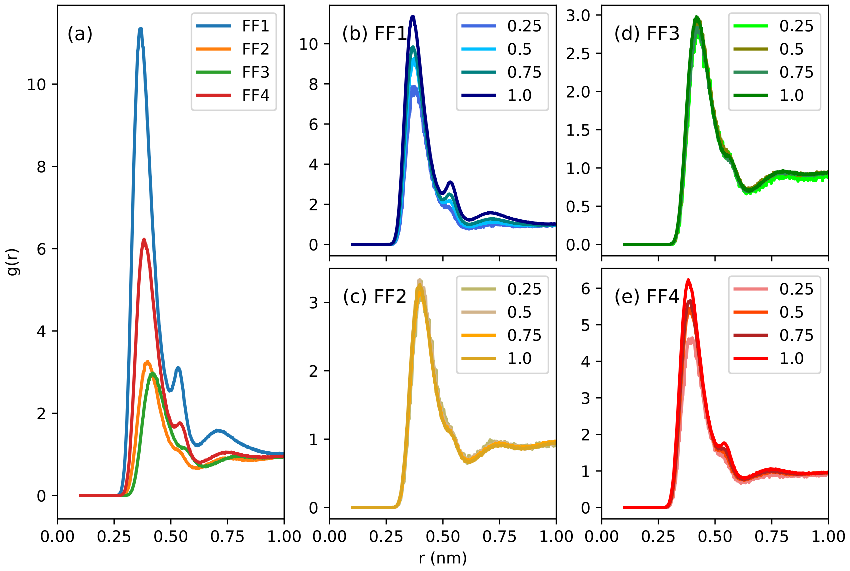

Figure S7: RDF for K^+-K^+ as a function of a) Force field (for 1.0 mol/kg KOH at 333K); and KOH concentration (at 333K) in b) FF1 c) FF2 d) FF3 e) FF4

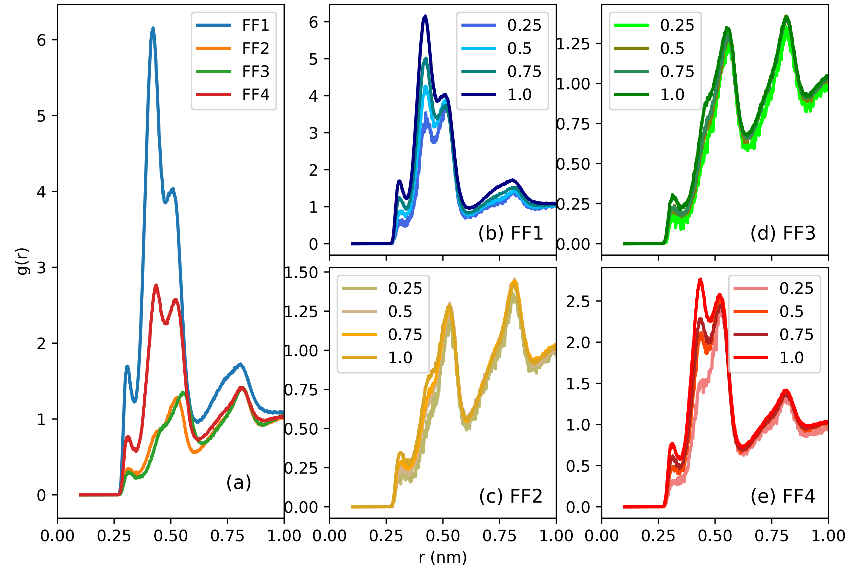

Figure S8: RDF for OH^--OH^- as a function of a) Force field (for 1.0 mol/kg KOH at 333K); and KOH concentration (at 333K) in b) FF1 c) FF2 d) FF3 e) FF4

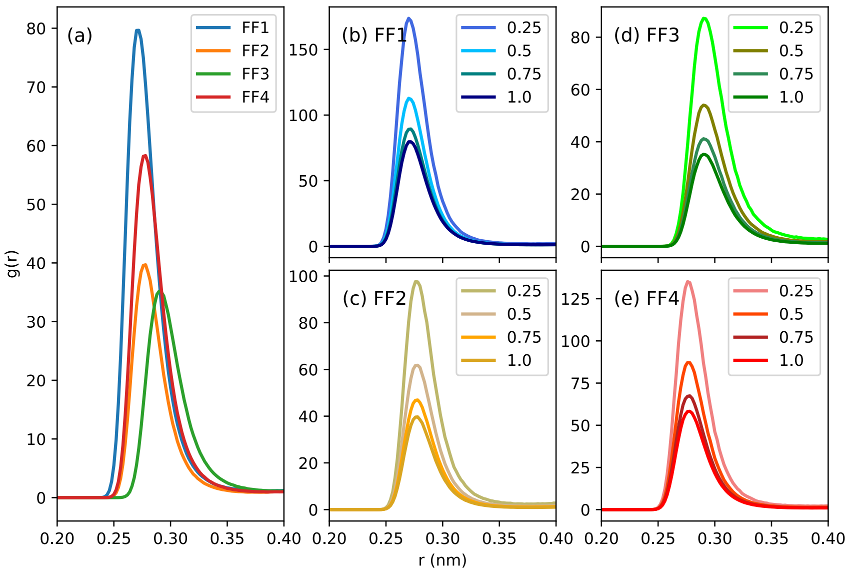

Figure S9: RDF for K^+-OH^- as a function of a) Force field (for 1.0 mol/kg KOH at 333K); and KOH concentration (at 333K) in b) FF1 c) FF2 d) FF3 e) FF4

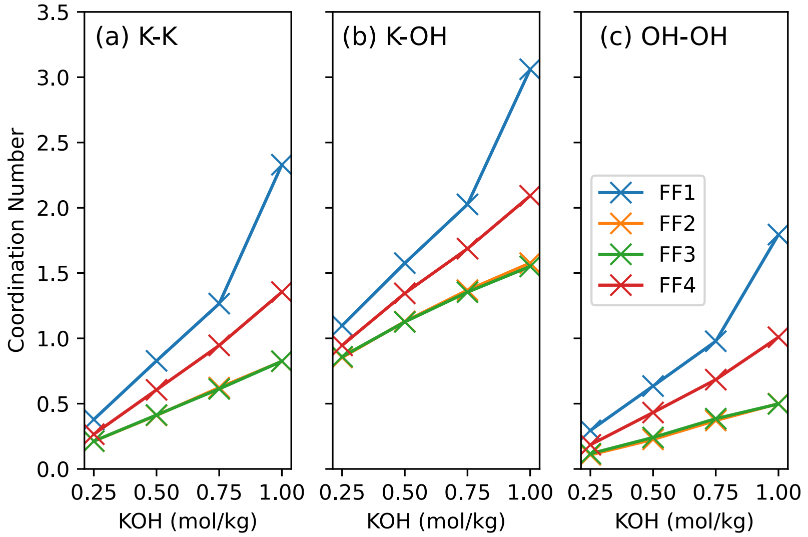

Figure S10: Coordination numbers as a function of KOH concentrations and force fields (at 333K) for a) K-K b) K-OH c) OH-OH

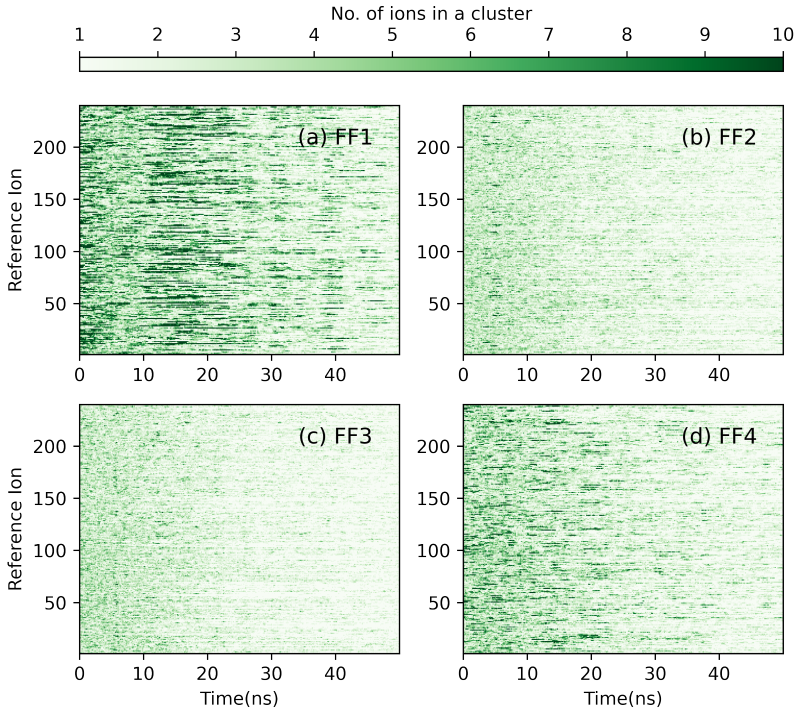

Figure S11: Total number of ions in a cluster as a function of time in 1.0 mol/kg KOH (at 333K) as predicted by a) FF1 b) FF2 c) FF3 d) FF4

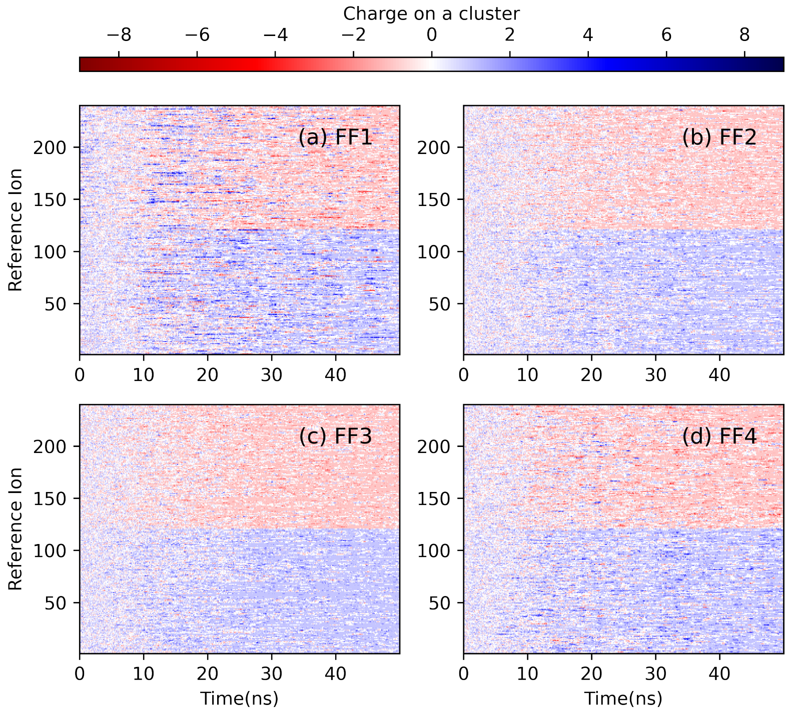

Figure S12: Total charge on a cluster as a function of time in 1.0 mol/kg KOH (at 333K) as predicted by a) FF1 b) FF2 c) FF3 d) FF4

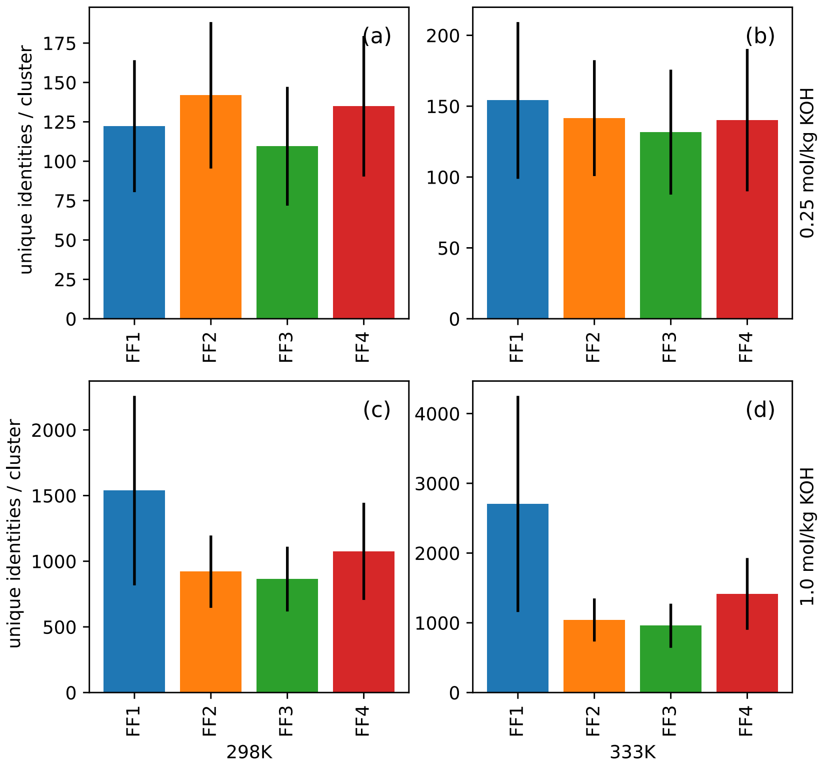

Figure S13: Number of unique identities per cluster in different force fields. Top row: 0.25 mol/kg KOH at 298K (a) and 333K (b). Bottom row: 1.0 mol/kg KOH at 298K (c) and 333K (d) respectively.

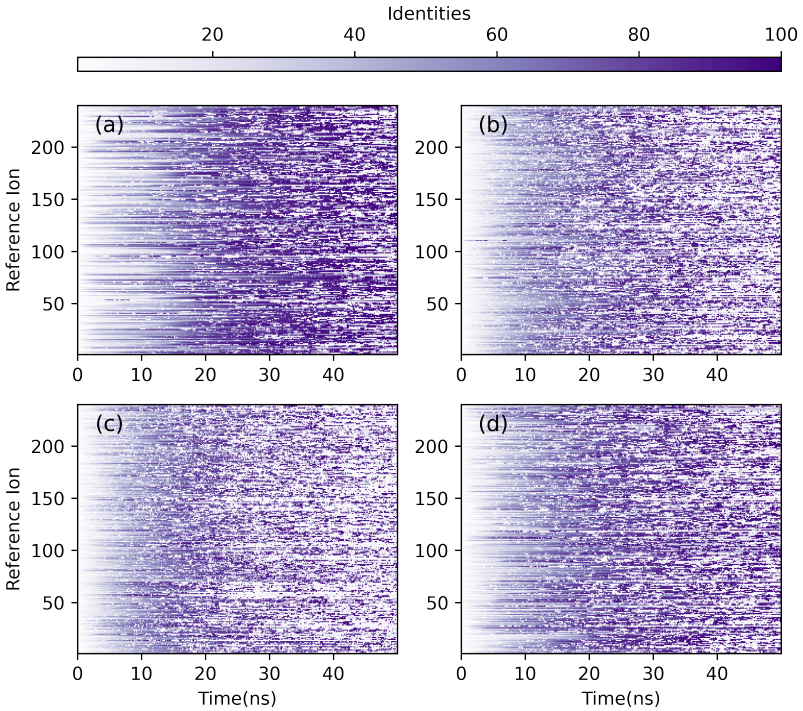

Figure S14: Identities assumed over time in 1.0 mol/kg KOH (at 333K) as predicted by a) FF1 b) FF2 c) FF3 d) FF4

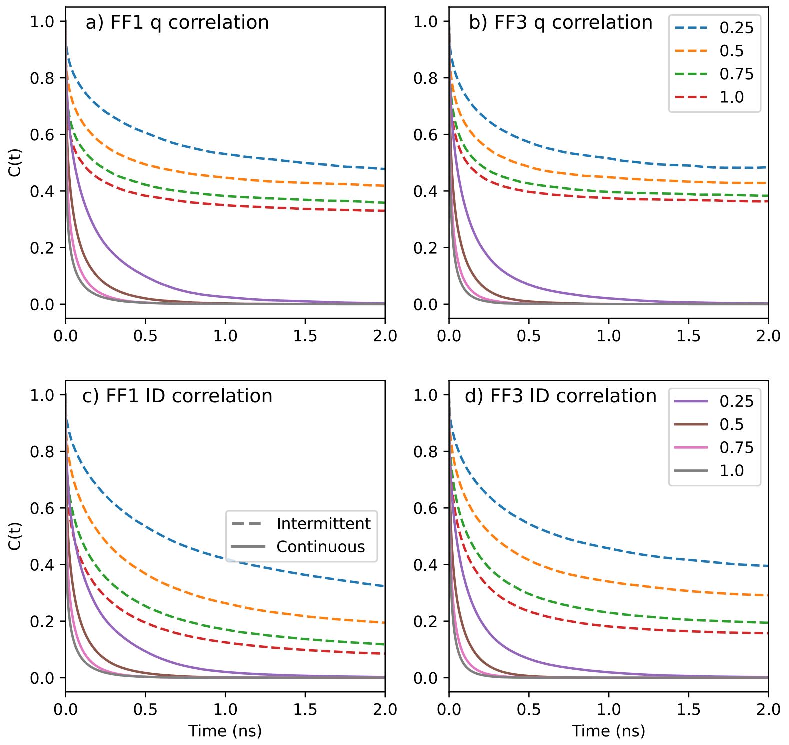

Figure S15: Charge and identity correlations in FF1 (a and c) and FF3 (b and d)
